# Implementation of a portable device for real-time ECG signal analysis

**DOI:** 10.1186/1475-925X-13-160

**Published:** 2014-12-10

**Authors:** Taegyun Jeon, Byoungho Kim, Moongu Jeon, Byung-Geun Lee

**Affiliations:** School of Information and Communications, Gwangju Institute of Science and Technology, Gwangju, Republic of Korea; School of Mechatronics, Gwangju Institute of Science and Technology, Gwangju, Republic of Korea; Broadcom Corporation, Irvine, CA 92617 USA

**Keywords:** Portable ECG device, Heart disease, Atrial fibrillation, Myocardial ischemia, Feature extraction, Embedded device

## Abstract

**Background:**

Cardiac disease is one of the main causes of catastrophic mortality. Therefore, detecting the symptoms of cardiac disease as early as possible is important for increasing the patient’s survival. In this study, a compact and effective architecture for detecting atrial fibrillation (AFib) and myocardial ischemia is proposed. We developed a portable device using this architecture, which allows real-time electrocardiogram (ECG) signal acquisition and analysis for cardiac diseases.

**Methods:**

A noisy ECG signal was preprocessed by an analog front-end consisting of analog filters and amplifiers before it was converted into digital data. The analog front-end was minimized to reduce the size of the device and power consumption by implementing some of its functions with digital filters realized in software. With the ECG data, we detected QRS complexes based on wavelet analysis and feature extraction for morphological shape and regularity using an ARM processor. A classifier for cardiac disease was constructed based on features extracted from a training dataset using support vector machines. The classifier then categorized the ECG data into normal beats, AFib, and myocardial ischemia.

**Results:**

A portable ECG device was implemented, and successfully acquired and processed ECG signals. The performance of this device was also verified by comparing the processed ECG data with high-quality ECG data from a public cardiac database. Because of reduced computational complexity, the ARM processor was able to process up to a thousand samples per second, and this allowed real-time acquisition and diagnosis of heart disease. Experimental results for detection of heart disease showed that the device classified AFib and ischemia with a sensitivity of 95.1% and a specificity of 95.9%.

**Conclusions:**

Current home care and telemedicine systems have a separate device and diagnostic service system, which results in additional time and cost. Our proposed portable ECG device provides captured ECG data and suspected waveform to identify sporadic and chronic events of heart diseases. This device has been built and evaluated for high quality of signals, low computational complexity, and accurate detection.

## Background

Heart disease is one of the major causes of death, especially for the elderly population in many countries. A total of 42 million out of 84 million people in North America who have one or more cardiovascular diseases are estimated to be older than 60 years old [1]. The existing ambulatory ECG monitoring systems take a considerable amount of time and effort, record ECG signals in patients through long-term hospitalization, and the ECG data have to be sent to professionals for diagnostic analysis. However, a portable ECG device, which provides real time monitoring of heart disease, can help medical decision making by detecting sporadic events of heart disease as early as possible. If the patient with chronic diseases worn a ECG device without any real time monitoring function, the primary defect of such solution is arise from lack of help when a major incident occurs during the monitoring. The device without real time analysis recorded ECG waveform but no immediate response is taken to help the patient. The device with real time analysis can support medical decision with captured ECG waveform during doubtful sections of incident as a black box. Therefore, a portable ECG device is required for monitoring and identification of sporadic and chronic events of heart diseases.

**Figure 1:**
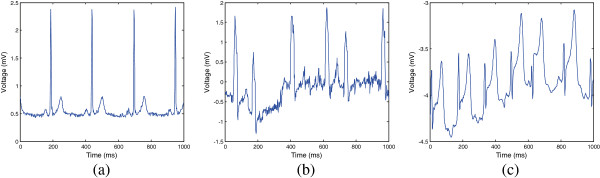
**ECG signals.** Examples of ECG signals in various cases. **(a)** Normal ECG, **(b)** irregular ECG containing atrial fibrillation, and **(c)** ST segment elevation containing myocardial ischemia.

Representative ECG signals of a normal ECG, in atrial fibrillation (AFib), and in myocardial ischemia, are shown in Figure 1. AFib, which is caused by a rapid and irregular heart beat at a rate of 400 to 600 beats per minute, is a type of arrhythmia [2–5]. AFib can be detected by monitoring the heart beat and absence of the P wave. Myocardial ischemia, caused by blockage of coronary arteries, reduces oxygen supply from the heart [6–9], and can be detected by monitoring abnormal divergence in the PR and ST segments. Even though various detection methods have been proposed for AFib and myocardial ischemia [10–17], they can only detect a single disease. To simultaneously detect AFib and ischemia, a compact and efficient architecture for detecting heart disease is required.

Developing a portable ECG monitoring device has been an active focus of research (Table 1). Most of the portable ECG device have simple metal contacts that the user can place their thumbs or other fingers on or place against bareskin, such as on the chest [18–26]. The metal contacts are much more convenient and faster to use than adhesive skin electrodes. In general, there are more artifact noise and artifacts called baseline wander in the typical thumb contact. On the other hand, recordings using adhesive electrodes are much cleaner, consistent and more accurate [27–31]. While most of these devices acquire and record ECG signals, they do not provide real-time identification for analysis of heart disease. Signal analysis of two devices is below the level that recognize existence and nonexistence of irregular rhythm trends [19, 21]. From a supporting medical analysis perspective, professional reports from the interpretation center are provided as medical analysis service with extra charge [18, 21, 22, 25, 27, 29, 30]. Simple information of heart rate and irregularity is provided [19, 20, 23, 24, 26, 28]. Thus, it is important to develop new healthcare device to achieve meaningful monitoring and real-time alert system.

**Table 1:** **Reviewed existing ECG devices and their properties for ECG signal analysis**

Product	Contact	Software for ECG	Support for medical analysis
iPhoneECG [18]	Dry metal for fingers	Measurement	Professional report
Smartheart [27]	Adhesive electrodes	Measurement	Interpretation center
CardioDefender [19]	Dry metal for fingers	Signal analysis	Irregularity detection
ME 80 [20]	Dry metal for fingers	Measurement	Heart rate calculation
ELI 10 mobile [28]	Adhesive electrodes	Measurement	ECG interpretation
EPI Life [21]	Dry metal for fingers	Signal analysis	Interpretation center
ReadMyHeart [22]	Dry metal for fingers	Measurement	Professional report
ECG Check [23]	Dry metal for fingers	Measurement	Heart rate calculation
Dicare-m1CP [24]	Dry metal for fingers	Measurement	Irregularity detection
HeartCheck PEN [25]	Dry metal for fingers	Measurement	Professional report
MD100E [29]	Adhesive electrodes	Measurement	Professional report
PC-80 [30]	Adhesive electrodes	Measurement	Professional report
REKA E100 [26]	Dry metal for fingers	Measurement	ECG interpretation
EKG/ECG-80A [31]	Adhesive electrodes	Measurement	Built-in ECG printer

Also, several classification methods are implemented for cardiac disease detection. We already validated that SVM has outperformed against kernel density estimation and artificial neural networks as classifier in previous work [16, 17]. Principal component analysis (PCA), Genetic algorithm (GA), rule-based methods are also adapted to detect cardiac diseases. However, the platforms of these classifiers are limited to desktop and laptop. Thus, these classifiers are insufficient to work in real-time on mobile and portable platform [32]. In order to overcome all these weakness, this study aimed to implement a portable real-time ECG processing device with an algorithm for detecting heart disease based on the feature extractors reported in previous studies [16, 17].

## Methods

### Overall framework

**Figure 2:**
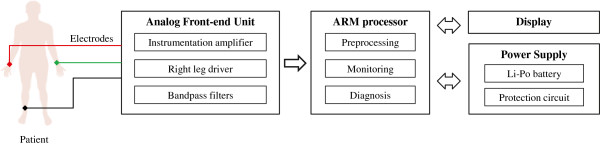
**Overall framework.** Overall framework configuration of the proposed portable ECG device.

As shown in Figure 2, the proposed portable ECG device was designed using the following blocks: a simplified analog front-end, an ARM processor to realize signal monitoring and analysis, an interactive display unit, and a power source. The features of the individual blocks are as follows below.

- *Reduced hardware complexity:* We aimed to develop a powerful software working platform using an ARM processor to simplify the hardware requirements. Consequently, portability of the device can be accomplished. The minimization analog front-end was realized by implementing most of the analog functions (highpass, lowpass, and notch filters) using digital filters. In addition to device compatibility, this also helped to reduce power consumption of the device and extended battery life.
- *Real-time processing:* Computational complexity is one of the major obstacles while implementing hardware in real-time. However, to alleviate this drawback, the proposed software was modified and implemented in an ARM processor [16, 17]. Furthermore, the digital filter removes various types of noises and baseline wander from the preprocessed ECG data and then it tends to extract and classify the features from the filtered ECG data for analysis. The processed ECG data and results of the analysis can be displayed using an interactive LCD display. To summarize the analysis results, the device reports averaged feature values and detected diseases every minute.
- *Simultaneous feature extraction for AFib and ischemia:* We considered two distinct diseases corresponding to atrial and ventricular activity. To simultaneously describe heart activity, we implemented features for irregularity, shape, area, slope, and distribution of ECG data [16, 17]. Feature extraction methods to represent irregularity were simplified without compromising detection performance. Furthermore, the extracted features from the ECG signal were classified into AFib and myocardial ischemia. Therefore, if target diseases are changed or added, we can easily adjust the feature extractors and train the classifiers accordingly.

### Analog front-end

ECG signals that are generated by electrical activity in the heart are a small pulse train of which the amplitude is less than 2 mV and bandwidth ranges from 0.05 to 150 Hz. Because the ECG signal is often corrupted by various noises originating from the body and analog signal processing hardware, efforts have been made to capture a clean ECG signal with error-prone analog circuits, such as amplifiers and filters [33–35]. In the current study, analog signal processing was minimized by realizing most of the signal processing in the software using a cheap and general purpose ARM processor to enable a small system for portability and reconfigurable for use in various conditions. The analog front-end has two major functions (1) amplifying the ECG signal to be sampled and quantized properly by an analog-to-digital converter (ADC), and (2) attenuating high-frequency noises that can corrupt the sampled ECG signal because of an aliasing effect. For the sake of cost reduction and simplicity of hardware, an ADC embedded in the ARM processor was used instead of using an additional high-performance ADC. An ADC running at a sampling frequency (*F*_*S*_) of 1 kHz has a 12-bit resolution with a reference voltage (*V*_*REF*_) of 1.8 V.

**Figure 3:**
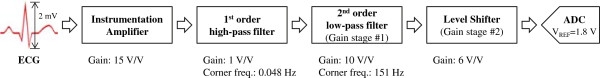
**H/W block diagram.** Hardware block diagram of the proposed minimized analog front-end.

Figure 3 shows a block diagram of the analog front-end. The analog front-end contains an amplifier with a first-order high-pass filter for DC or offset rejection, two gain stages with a level shifter, and a second-order low-pass filter (LPF) as an anti-aliasing filter. Because the peak ECG signal has an amplitude of approximately 2 mV, the required gain is 900 (V/V), and this is implemented by spreading it over the gain stages. The amplifier has a gain of 15 V/V and the two gain stages are 10 and 6 V/V.

### ECG signal processing and learning on the ARM processor

**Figure 4:**
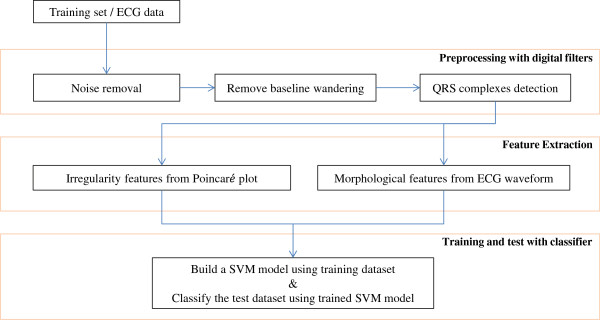
**S/W block diagram.** Block diagram of the proposed software for training and test phases.

The proposed software has two different operating modes of the training phase and test phase. A schematic setup of the proposed software is shown in Figure 4. The software can be divided into three functional blocks, including preprocessing, feature extraction, and classification. At the preprocessing stage, the noise and baseline wander of the measured ECG data were removed. We simultaneously labeled the locations of the QRS complex, P wave, and T wave by using the QRS complex detector. Later, using the labeled QRS complex, we calculated interbeat intervals and created Poincaré plots. In the feature extraction process, we extracted feature values for irregularity and morphological shape from a sliding window. Eventually, at the classification block, we built a trained support vector machine (SVM) model that could detect heart disease from the test data. Furthermore, the trained SVM model was moved to the ARM processor and operated to classify heart disease based on the test phase. In this study, we trained our proposed system to detect AFib based on the MIT-BIH AF, Arrhythmia, CinC 2001, and CinC 2004 databases, and to detect myocardial ischemia using the European ST-T databases [36]. Primarily, the training phase was conducted to train our proposed SVM model, by exploiting these databases. Conversely, the test phase provided the analytical results of the measured ECG signals that were acquired using the proposed electrodes.

### Preprocessing with digital filters

As mentioned above, in addition to filtering provided at the analog-front end, ECG data are processed by digital filters to further reduce the noise and interference, which are caused by the following artifacts: contraction of skeletal muscle, fluctuation of the power supply, and mechanical force on electrodes. The digital filters have the advantage of being able to easily change the filter characteristics (i.e., modification of filter coefficients). Therefore, to remove accumulated noise, we combined 0.05-Hz highpass, 60-Hz notch, and 40-Hz lowpass filters using Butterworth filters, which are a type of infinite impulse response. Advantages are that the implemented filters require a small number of operations per time step.

Similarly, to remove baseline wandering effects, we decomposed the signal vectors using a wavelet operator, which has a similar shape as QRS complexes. In this step, we used Daubechies8 as mother wavelet since Daubechies wavelet family have similar shapes with QRS complex. Measured ECG data is decomposed into eight levels with detail and approximation coefficients. The sequences of detail coefficients represent prominent points and segments, and the sequences of approximation coefficients represent an unexpected wandering baseline. Therefore, important sequences of detail coefficients can be retained from the input ECG data. Subsequently, to detect QRS complexes, we assigned proper locations of QRS complexes using the wavelet scale selection method. From the decomposed coefficients, we substituted the zero-vector to all sequences, except for one detail coefficient sequence. By repeating this step to all acquired sequences of detail coefficients, we measured the score to find the protruding segment. The QRS complex was then assigned as *QRS*_*i*_ by maximum protruding values for each target segment. And the interbeat interval *I*_*i*_ is RR interval which is calculated from the difference of two consecutive QRS complexes as follows: *I*_*i*_=*QRS*_*i*_−*QRS*_*i*−1_. More detailed descriptions for detecting QRS complexes have been previously reported [17].

### Feature extraction and classifications

We used sliding windows of size 10 seconds and included about 10 consecutive interbeat intervals. The sliding window continuously moves to the next interbeat interval, overlapping the half of the interval. Therefore, the features for irregularity and morphological shape can be extracted from the sliding window. To represent irregularity on ECG data, we created a Poincaré plot, which showed self-similarity in periodic functions and sequences. A point in the plot can be defined as *P*_*k*_=(*I*_*k*_,*I*_*k*+1_), where *I*_*k*_ is k-th interbeat interval. If the measured points converge near to a central point, this phenomenon implies the interbeat intervals are almost the same in the observed sliding window. In contrast, a pattern with diffused points represents irregular interbeat intervals.

In real-time processing, to represent irregularity to detect AFib, we modified three features based on our previous study [16]: (1) a simplified mean stepping increment, (2) the sum of the distance from the major interbeat interval point, and (3) the number of clusters in a Poincaré plot. These features are extracted from the current sliding window which contains *n* interbeat intervals. The first feature is a simplified mean stepping increment. The distance between two consecutive points *P*_*k*_ and *P*_*k*+1_ in the plot using the Euclidean distance can be formulated as follows: . If the two consecutive points are regular beats, the distance converges to zero. However, irregularity of ECG signals is accumulated with increasing distances. Furthermore, by removing the common points, the simplified value of the summation can be implemented in the portable device. The simplified version of mean stepping increment was modified as follows:
1

The second feature is the sum of the distance from a diagonal line in a Poincaré plot. If a point is located around a diagonal line in the plot, then it denotes that x and y positions have similar values. This characteristic also means that interbeat intervals are regularly generated. This dispersion feature illustrates how to distribute the points in a plot from regular interbeat intervals as follows:
2

The third feature is the number of clusters in a Poincaré plot, and this is decided from the spectral clustering method [37]. In normal cases, the interbeat intervals are regular. Therefore, the corresponding points in the Poincaré plot are grouped as a small cluster, i.e., closely located points. After the clustering process, a plot of normal ECG signals shows a consistent group of points. However, a plot of AFib shows scattered points.

To capture characteristics related to myocardial ischemia, we focused on the shape of the ST segment and QRS complexes. We can extract significant morphological information through QRS complexes and the T wave peak. The first feature of ischemia is cumulative voltage values, which measure how the T wave is elevated from a normal QRS onset point. If the ST segment deviates from normal levels, this feature value is highly increased. The mean value of the ST segment is usually located at around the QRS onset point. The second and third features are a voltage deviation in the ST segment and a slope from the QRS onset to the offset point, respectively.

As explained above, at the training phase, feature values are agglomerated together to a feature space. At the test phase, extracted features from preprocessed ECG signals are classified using support vector machines every minute. We classified the extracted test feature values using the trained SVM model.

### Implementation and programming environment

The proposed device captures ECG signals from the human body using a four-pole clip electrode through the analog front-end. Furthermore, we used three-lead ECG signals from the left arm, right arm, and right leg. The size of the analog front-end module is (*H*,*W*,*D*)=(0.1 *cm*,3.5 *cm*,7.7 *cm*), which can easily be embedded into a wearable ECG acquisition device. The instrumentation amplifier (TI INA216) and the OP-Amp (LM358) are used to obtain regulated ECG signals from the human body. These circuits are suitable for a portable device with the data acquisition system.

**Figure 5:**
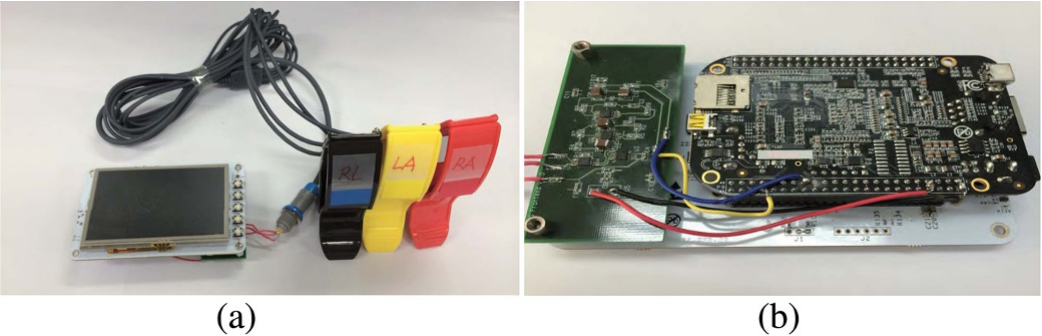
**Implemented hardware.** Photographs of the implemented hardware. **(a)** Prototype consisting of electrodes, display, analog front-end, and ARM processor. **(b)** Detail view of implemented device.

To provide portability, as well as an interactive service, we attached a compact (4.3” TFT) LCD display device on top of the ARM processor. The LCD4 display provides a simple and compact display solution with touch screen capability. This display offers a good resolution of 480×272 and a four-wire resistive touch screen provides the opportunity to design various types of graphic user interfaces. This system is equipped with a compact lithium polymer single cell battery with 1300 mAh and the battery should last for approximately 3 hours with a full charge. The hardware implementation is shown in Figure 5.

For scalability, we developed our software using C++ language and QT for embedded Linux. Preprocessing, feature extraction, classification, and graphic user interface for the software were implemented and tested in the ARM processor. The classifier was used based on libSVM, which provides an integrated library for support vector machines [38]. The software consists of three screen activities, including the main signal view, a Poincaré plot, and data description view.

**Figure 6:**
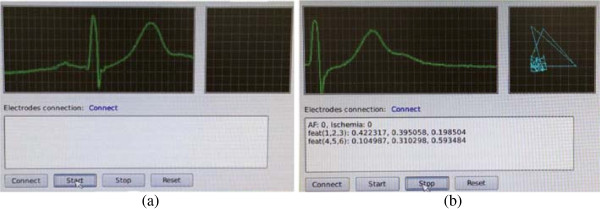
**Implemented software.** Real-time ECG monitoring on human subject. **(a)** Input ECG signals are visualized in the main view. **(b)** A Poincaré plot and extracted features are shown on the screen.

Figure 6 shows the ECG monitoring and real-time test results on human subjects. The proposed device simultaneously records ECG signals from a user, then displays it on the real-time screen. By collecting ECG signals continuously for one minute, we can then calculate inter-beat intervals for each QRS complex. Furthermore, the Poincaré plot is drawn from extracted inter-beat intervals and is updated after every minute. The plot shows heart activity by regular drawing of points. The activity of points converging on one centroid represents that heart activity is regular and normal. On the other hand, when the points are irregularly distributed, this plot represents one of the typical AFib cases [39, 40].

Additionally, the data description view is displayed after the measured ECG data are analyzed. The description view provides essential information with the average extracted feature values and classification results. The numbers with AF and ischemia represent the existence of corresponding diseases as (0 for nonexistence and 1 for existence). The numbers with “feat (1,2,3)” represent a set of three features for detecting AFib as follows: (1) simplified mean stepping, (2) the sum of the distance from the major interbeat interval point, and (3) the number of clusters in a Poincaré plot. Similarly, the numbers with “feat (4,5,6)” represent a set of three features for detecting myocardial ischemia as follows: (4) cumulative voltage value, (5) voltage deviation in the ST segment, and (6) slope from the QRS onset to the offset. Since the range of feature values varies widely, all ranges of features are normalized. The summarized information and corresponding ECG records would help medical decision for physicians.

## Results and discussion

The proposed device is characterized as follows: (1) it reduces hardware complexity, (2) it has real-time processing, and (3) it has simultaneous feature extraction for AFib and ischemia. To validate our device, we examined the quality of acquired signals, computational complexity, and the accuracy of the embedded algorithm.

### Performance evaluation of the analog front-end

We conducted an experiment to determine the quality of the measured ECG signals by comparing public ECG databases, synthesis signals, and collected ECG signals The purpose of this experiment was to determine whether our ECG measurement is similar to well-organized cardiac databases. We prepared three ECG signals as follows. First, existing records from the PhysioNet databases were chosen as public ECG signals [36]. Second, synthesized ECG signals with simulated random noise and wandering baseline were generated as follows:
3

The noise was added to the public ECG signals as Eq. 3. The waveform of our result had a similar amplitude to the public ECG signals and stable shapes. We then transformed signals from the time domain to the frequency domain. The measured ECG signals were similar to the quality of the public cardiac database. This finding indicates that various types of noises were well controlled and maintained for the purpose of analysis. We then estimated the power spectral density using a periodogram in the frequency domain. We calculated the similarity by using the root-mean-square-error (RMSE) between the signals as follows: *RMSE*(*ECG*_*existing*_,*ECG*_*simulated*_)=0.0308,*RMSE*(*ECG*_*existing*_,*ECG*_*our*_)=0.0545,*RMSE*(*ECG*_*simulated*_,*ECG*_*our*_)=0.0853. The similarity results showed that our device captured ECG signals that were as clean as the public cardiac database. Additionally, ECG signals from our device were distinguished from simulated ECG signals. This result indicates the quality of our analog front-end and digital filters.

In addition, We have conducted additional tests to compare the SNR before and after filtering using MIT-BIH Noise Stress Test Database [36]. This database provides three types of noises (e.g. baseline wander (BW), muscle artifact (MA), and electrode motion artifact (EM)). Various noisy signals have been generated and tested by combining normal ECG signal from MIT-BIH Arrhythmia database with the noises [36]. The following Table 2 shows the experiment results of comparing the SNR before and after the filtering. Average SNR improvements of BW, EM, and MA are (7.3632 dB, 5.2544 dB, 6.5382 dB), resulting in overall SNR improvement of 6.3853 dB.

**Table 2:** **Test results to compare the SNR before and after filtering**

Clean signal from	Type of	SNR before	SNR after	SNR
MIT-BIH Arrhythmia DB	noise	filtering (dB)	filtering (dB)	improvement (dB)
Record 118	BW	6	15.1689	9.1689
Record 118	EM	6	13.7682	7.7682
Record 118	MA	6	14.4875	8.4875
Record 118	BW	10	18.5482	8.5482
Record 118	EM	10	15.7853	5.7853
Record 118	MA	10	17.5477	7.5477
Record 118	BW	14	19.4872	5.4872
Record 118	EM	14	17.5643	3.5643
Record 118	MA	14	18.6872	4.6872
Record 119	BW	6	14.2548	8.2548
Record 119	EM	6	12.2346	6.2346
Record 119	MA	6	13.7458	7.7458
Record 119	BW	10	17.9658	7.9658
Record 119	EM	10	16.4867	6.4867
Record 119	MA	10	17.1054	7.1054
Record 119	BW	14	18.7548	4.7548
Record 119	EM	14	15.6875	1.6875
Record 119	MA	14	17.6561	3.6561
Average		10	16.3853	6.3853

### Performance evaluation of computational complexity

Real-time monitoring of the device was tested by measuring the computational time of the primary components. Computational complexity is based on processes, such as signal acquisition, digital filters, feature extraction, and classifications. We measured average values for the central processing unit (CPU) and memory use per minute. The results of the performance evaluation are as follows: (1) average CPU use: 33%, (2) minimum CPU use: 11%, (3) maximum CPU use: 56%, and (4) average memory consumption: 55%. Our results showed the feasibility of our device in real situations.

### Evaluation of sensitivity and specificity for AFib and myocardial ischemia

We also compared our results with recent detection algorithms such as Artificial Neural Networks, Principal Component Analysis, Genetic Algorithms, Rule-based method, and morphological analysis [10–17]. For each heart disease, we already found the set of parameters with the best classification results in our previous work [16, 17]. For the purpose of overall comparison, we evaluated our method with the following three sets of MIT-BIH databases: (1) MIT-BIH AF and arrhythmia, (2) CinC challenge 2001 and 2004 databases, and (3) European ST-T database from PhysioNet. The number of waveforms are (1) 48 and 27, (2) 300 and 110, and (3) 90, respectively. These waveforms randomly partitioned into 10 equal size sub-samples for 10-fold cross validation. Of the 10 sub-samples, a single sub-sample is retained as the validation data for testing the model, and the remaining 9 samples are used as training data. The cross-validation process is then repeated 10 times, with each of the 10 samples used exactly once as the validation data. Then, the 10 results from the folds are averaged to produce a single estimation result. To compare the classification results, we measured the sensitivity and specificity as follows:
4

Where true positive (TP) implies normal beats, which are correctly detected as annotated. False positive (FP) represents abnormal beats that are classified into normal labels. True negative (TN) denotes abnormal beats that are detected as annotated with heart disease. False negative (FN) indicates normal beats, which are considered as abnormal cases. Table 3 shows the results of the classification with sensitivity and specificity. The average sensitivity and specificity of our method were 95.1% and 95.9%, respectively. These results indicate that our method effectively detects AFib and ischemia cases with a higher performance of detection for distinct cardiac diseases, while existing methods only focus on one target disease.

**Table 3:** **Offline classification results for AFib and ischemia using an SVM**

Previous works	Target	Databases	Sensitivity	Specificity
	diseases			
Papaloukas et al. [10]	Ischemia	European ST-T	0.9	0.9
Goletsis et al. [11]	Ischemia	European ST-T	0.912	0.909
Exarchos et al. [13]	Ischemia	European ST-T	0.912	0.922
Park et al. [17]	Ischemia	European ST-T	0.957	0.953
Dash et al. [15]	AFib	MIT-BIH AF and MIT-BIH Arrhythmia	0.944	0.951
Logan and Glass [12]	AFib	MIT-BIH AF	0.96	0.89
Kikillus et al. [14]	AFib	MIT-BIH AF and MIT-BIH NSR	0.944	0.934
Park et al. [16]	AFib	CinC 2001 and 2004	0.914	0.929
This work	AFib	MIT-BIH AF and MIT-BIH Arrhythmia	0.956	0.962
	AFib	CinC 2001 and 2004	0.928	0.938
	Ischemia	European ST-T	0.969	0.977

## Conclusion

In this study, we proposed and implemented a portable ECG device for real-time and personal purposes. We reduced the hardware complexity by using the digital filter-driven hardware architecture. By using this device, patients can keep tracking the condition of their heart on a daily basis, at low cost. According to the experimental results with MIT-BIH databases, our algorithm has a higher sensitivity and specificity of 95.1% and 95.9%, respectively. In addition, the proposed device has lower computational complexity than other existing detection algorithms that capture abnormal heart activities from atrial and ventricular chambers on portable and mobile platform. In summary, our device contributes to excellent monitoring and acceptable analysis results for helping medical decision making. Our results provide empirical evidence to substantiate real-time as well as show that our portable personal health care device has high quality signals, low computational complexity, and accurate detection ability.
